# Polymethyl methacrylate microplastics affect oral microbiota diversity and *Streptococcus mutans* biofilm formation

**DOI:** 10.3389/fmicb.2026.1811667

**Published:** 2026-04-14

**Authors:** Bing Yang, Ziyv Wu, Xing Cui, Yingjie Yi, Faming Chen, Guofeng Wu

**Affiliations:** 1 Nanjing Stomatological Hospital, Affiliated Hospital of Medical School, Institute of Stomatology, Nanjing University, Nanjing, China; 2 State Key Laboratory of Oral & Maxillofacial Reconstruction and Regeneration, National Clinical Research Center for Oral Diseases, Shaanxi International Joint Research Center for Oral Diseases, Department of Periodontology, School of Stomatology, The Fourth Military Medical University, Xi’an, China

**Keywords:** dental caries, microplastics, oral microbiota, polymethyl methacrylate, *Streptococcus mutans*

## Abstract

**Introduction:**

Polymethyl methacrylate (PMMA)—a widely applied dental resin-based material—contributes to oral PMMA microplastics (PMMA-MPs) exposure through masticating. PMMA-MPs may facilitate pathogen adhesion, posing substantial risks to oral health. Dental caries represents the most prevalent chronic infectious oral disease, characterized by progressive lesions that may induce pain, tooth structure loss, and compromised masticatory efficiency. *Streptococcus mutans* have been widely identified as the primary etiological agents responsible for caries pathogenesis. This study aimed to investigate the effects of PMMA-MPs on oral microbiota closely associated with dental caries.

**Methods:**

The impacts of PMMA-MPs were assessed using a standardized murine oral exposure model, followed by the quantification of PMMA-MPs-associated shifts in oral microbiota using high-throughput 16S rRNA gene sequencing. Compared with the control group, PMMA-MPs significantly altered the diversity of oral microbial communities in mice, with a notable increase in the relative abundance of *Streptococcus*. Based on the 16S sequencing results, *S. mutans* was selected for subsequent *in vitro* experiments.

**Results:**

PMMA-MPs markedly enhanced the growth, biofilm formation, and virulence factor synthesis of *S. mutans*. Transcriptomic analysis revealed that PMMA-MPs may promote biofilm formation through pathways including ABC transporters, quorum sensing, and purine metabolism. Additionally, PMMA-MPs exposure enhanced bacterial antibiotic tolerance.

**Discussion:**

Overall, our results revealed that PMMA-MPs can alter the composition of the oral microbial community, while enhancing both the virulence factors and antibiotic tolerance of *S. mutans* biofilms.

## Graphical abstract

**Figure:**
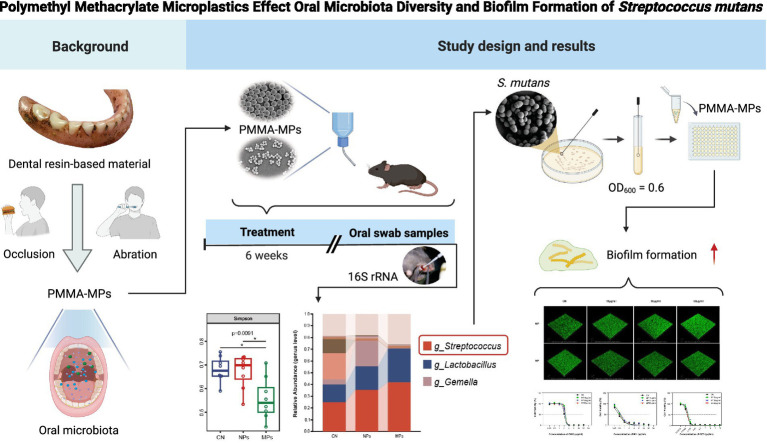

## 1 Introduction

Globally, approximately 2.43 billion people are affected by dental caries to varying degrees (Vos et al., 2012). *Streptococcus mutans* have been widely identified as the primary etiological agents responsible for caries pathogenesis (Simón-Soro and Mira, 2015). Biofilm formation in sucrose-containing environments is a critical factor in the cariogenicity of *S. mutans* (Lemos et al., 2019). *S. mutans* facilitates biofilm formation on the enamel surface by producing various glycosyltransferases that synthesize extracellular polysaccharides (EPS). Subsequently, it metabolizes various carbohydrates into organic acids through glycolysis and releases them into the biofilm, lowering the pH on the enamel surface (Krzyściak et al., 2014). Enamel demineralisation begins when the pH drops to 5.5 (Lin et al., 2021). In particular, *S. mutans* shows more extensive accumulation on resin-based composite surfaces, such as dentures, than on enamel (Svanberg et al., 1990), consequently elevating the caries risk in denture-wearing populations.

Biofilms are highly structured membranous complexes formed by bacterial communities that adhere to various solid surfaces and are embedded in a self-produced matrix of macromolecules, including exopolysaccharides, matrix proteins, and extracellular DNA (Tuon et al., 2022). Bacteria embedded within biofilms exhibit increased resistance to adverse environmental conditions and can evade host immune responses (Guo et al., 2023). Notably, Microplastics (MPs) can promote bacterial biofilm formation (Xu et al., 2019). Borcherding et al. (2014) reported that small-sized iron oxide nanoparticles enhance bacterial growth and induce biofilm formation. Similarly, Huang et al. (2024) found that polystyrene nanoplastics (NPs) at certain concentrations significantly promote bacterial biofilm formation. Furthermore, Biofilms are recognized as reservoirs of antibiotic resistance genes (ARGs) and a significant source of many multidrug-resistant bacteria (Olsen, 2015). MPs may promote mutations conferring antibiotic resistance in bacteria and facilitate the horizontal transfer of ARGs, thereby accelerating the emergence of multidrug-resistant strains (Ning et al., 2022). MPs foster unique ecological niches for biofilm formation and the proliferation of diverse microorganisms owing to their hydrophobic nature and large specific surface area, forming a distinct habitat known as the ‘plastisphere’ (Sun et al., 2023). Plastisphere in aquatic and terrestrial environments harbors diverse potential pathogens and ARGs, exhibiting a significant public health challenge (Junaid et al., 2022; Xu et al., 2022). The oral cavity shares similarities with aquatic environments in that it contains abundant fluid (saliva) and harbors diverse microbial communities. Furthermore, as a primary gateway for microplastic ingestion in humans, it remains unclear whether microplastics in the oral cavity may form a plastisphere that could potentially alter oral microbiota diversity.

Polymethyl methacrylate (PMMA)—a commonly used dental resin-based material for dentures—generates high concentrations of plastic particles during high-speed grinding procedures in clinical practice. Furthermore, PMMA undergoes continuous wear and leaching through routine oral physiological activities such as masticating, facilitating the release of MPs (Burian et al., 2021). Due to their widespread use and environmental release, PMMA-MPs have been detected in diverse environmental compartments (Mahadevan and Valiyaveettil, 2021) and human tissues, including liver, heart, and blood samples, indicating extensive human exposure (Saha et al., 2025). PMMA-MPs generated during dental procedures exhibit biotoxicity toward oral keratinocytes and trigger the expression of macrophage-associated inflammatory factors (Chen et al., 2025). Additionally, PMMA-MPs accumulation in the colon induces significant alterations in gut microbiota composition and increases microbial diversity (Zheng et al., 2025). However, research on the effects of PMMA-MPs on oral microbiota diversity and *S. mutans* remains limited.

Most existing studies on MPs and microorganisms have focused on environmentally derived particles, such as polystyrene, polyethylene, and polyvinyl chloride (Chen et al., 2022; Zhang et al., 2024; Sun et al., 2021), with limited focus on the correlation between PMMA-MPs and *S. mutans*. Specifically, the microbial effects of PMMA-MPs released into the oral environment, particularly on *S. mutans*, have not been thoroughly investigated. Therefore, this study aimed to explore the effects of PMMA-MPs on the biofilm formation and virulence of *S. mutans*.

## 2 Materials and methods

### 2.1 PMMA-MPs characterization

Based on the particle sizes of PMMA-MPs identified in human samples and existing toxicity data (Da Silva Brito et al., 2023; Ferrante et al., 2024; Song et al., 2024; Trifuoggi et al., 2019), particles with diameters of 20 μm and 80 nm were selected for this study. All PMMA-MPs were purchased from Beijing Zhongke Keyou Technology Co., Ltd. (Beijing, China). The surface morphology of the PMMA-MPs was observed using a scanning electron microscope (SEM; Regulus8200, HITACHI, Tokyo, Japan), and the particle size distribution was statistically analyzed. The zeta potential was measured using a zeta potential analyzer (Zetasizer Nano ZSE, Malvern, United Kingdom).

### 2.2 Establishment of animal models and 16S rRNA sequencing of oral microbiota

Thirty (4-week-old) male specific pathogen-free C57BL/6 mice were purchased from Zhejiang Vital River Laboratory Animal Technology Co., Ltd., Zhejiang, China (license key: SCXK (Zhe) 2020–0002). All experimental procedures were approved by the Institutional Animal Care and Use Committee (Approval number: IACUC – D2303113). After 1 week of acclimatization, the mice were randomly divided into three groups (*n* = 10 per group). The control group was administered sterile water, whereas the remaining two groups were exposed to sterile water containing PMMA particles: PMMA 80 nm (200 μg/mL) and PMMA 20 μm (200 μg/mL). All groups were fed the same diet. Following 6 weeks of continuous treatment, sampling was performed at least 2 h after the last food or water intake. Oral microbiota samples were collected from mice by wiping the buccal and lingual surfaces of all molars, as well as from occlusal surfaces, and the buccal mucosa with sterile swabs for subsequent 16S rRNA gene sequencing. Total bacteria DNA samples were extracted using the MagBeads FastDNA Kit for Soil (116564384) (MP Biomedicals, CA, United States). Following the manufacturer’s instructions, and stored at −20 °C prior to further analysis. The quantity and quality of extracted DNAs were measured using a NanoDrop NC2000 spectrophotometer (Thermo Fisher Scientific, Waltham, MA, United States) and agarose gel electrophoresis, respectively. PCR amplification of the bacterial 16S rRNA genes V4–V5 region was performed. PCR amplicons were purified with Vazyme VAHTSTM DNA Clean Beads (Vazyme, Nanjing, China) and quantified using the Quant-iT PicoGreen dsDNA Assay Kit (Invitrogen, Carlsbad, CA, United States). After the individual quantification step, amplicons were pooled in equal amounts, and pair-end 2,250 bp sequencing was performed using the Illlumina NovaSeq platform with NovaSeq 6,000 SP Reagent Kit (500 cycles) at Shanghai Personal Biotechnology Co., Ltd. (Shanghai, China). The α-diversity, β-diversity, species composition analysis and species difference analysis were analyzed on the online platform of Personalbio Genescloud^1^. The specific genes of *S. mutans* in each sample group was detected by qRT-PCR. Three independent experiments were performed. The primer sequences used in the experiment are listed in Supplementary Table S1.

### 2.3 Bacterial strains and growth curve analysis

The *S. mutans* strain UA159 was obtained from Nanjing Stomatological Hospital. The bacterial strains were cultured in brain heart infusion (BHI; Hope Bio-Technology Co., Ltd., Qingdao, China) broth medium at 37 °C under anaerobic conditions with 5% CO₂ for 48 h. Single colonies were then inoculated into fresh BHI liquid medium. When the bacterial culture reached the logarithmic growth phase (OD₆₀₀ = 0.6), the suspension was adjusted to 1 × 10^7^ CFU/mL for subsequent experiments. Biofilms were formed in BHI with 1% sucrose (1% BHIS).

PMMA particle suspensions were prepared in BHI broth at concentrations of 15, 30, and 60 μg/mL. For each experimental condition, 100 μL of PMMA particle suspension was mixed with 100 μL of bacterial culture (1 × 10^7^ CFU/mL) in 96-well plates, with three replicate wells per concentration. Two control groups were established: (i) a negative control consisting of 100 μL BHI broth mixed with 100 μL bacterial suspension, and (ii) a blank control containing 200 μL BHI broth alone. The plates were incubated for 24 h. Bacterial growth was monitored by measuring OD₆₀₀ every 2 h using a multifunctional microplate reader (SpectraMAX M3, Molecular Devices, United States). To account for potential interference from the culture medium, the OD values of the blank control were subtracted from the corresponding experimental values at each time point. Growth curves were then generated by plotting the corrected OD values versus time.

### 2.4 Quantification of biofilm formation

Biofilm formation following PMMA-MPs exposure was assessed by crystal violet staining. A 250 μL aliquot of bacterial suspension was mixed with an equal volume of 1% BHIS broth (containing either 0, 15, 30, or 60 μg/mL PMMA-MPs) in 24-well plates. Each concentration was tested in triplicate wells. Following 24 h incubation at 37 °C under anaerobic conditions (5% CO₂), the culture medium was carefully aspirated and replaced with fresh 1% BHIS broth. The biofilms were then cultured for an additional 24 h to achieve maturation (total incubation period: 48 h). The supernatant was carefully aspirated, and the biofilms were gently washed three times with phosphate-buffered saline (PBS; pH 7.4). The samples were then fixed for 15 min, air-dried, and stained with 0.1% (w/v) crystal violet for 15 min. After removing the staining solution, the biofilms were washed three times with PBS to remove unbound dye. The bound crystal violet was solubilized with absolute ethanol, and the OD₅₉₅ was measured using a microplate reader.

The thickness of bacterial biofilms following PMMA-MPs exposure was analyzed using confocal laser scanning microscopy (CLSM; ECLIPSE Ti2, Nikon, United States). A 500 μL bacterial suspension was mixed with an equal volume of 1% BHIS broth in 35-mm confocal dishes, with or without PMMA-MPs at concentrations of 15, 30, or 60 μg/mL. Each concentration was tested in triplicate. Following 24 h incubation at 37 °C, the culture medium was replaced with fresh 1% BHIS broth and incubated for 48 h to allow biofilm maturation. The supernatant was carefully removed, and the biofilms were gently washed with physiological saline. Biofilms were then stained with 1% SYTO (KGE2503-500, KeyGEN, Nanjing, China) for 15 min at 25 °C. Biofilm thickness was examined using CLSM at 60× magnification, and fluorescence intensity was quantified using Image-Pro Plus software.

### 2.5 Effects of PMMA-MPs on bacterial biofilm architecture

The impact of PMMA-MPs on bacterial biofilm morphology was examined using a SEM. Bacterial biofilms were cultured by inoculating 250 μL of bacterial suspension with an equal volume of 1% BHIS broth, with or without PMMA-MPs, in 24-well plates. Each concentration was tested in triplicate. Following 24 h of incubation at 37 °C, the medium was replaced with fresh 1% BHIS broth, and biofilms were allowed to mature for 48 h. Mature biofilms were gently washed three times with PBS and fixed with 2.5% glutaraldehyde at 4 °C for 12 h. After removing the fixative, samples were rinsed three times with PBS and dehydrated using a graded ethanol series before being air-dried. The prepared biofilms were mounted on monocrystalline silicon wafers, sputter-coated with gold, and imaged under a SEM.

### 2.6 EPS and acid production

The relative yield of EPS in biofilms was determined using the phenol–sulfuric acid assay. Bacterial biofilms were cultured by combining 100 μL of bacterial suspension with an equal volume of 1% BHIS broth in 96-well plates, with or without PMMA-MPs. Each concentration was prepared in triplicate. Following 24 h of incubation at 37 °C, the supernatant was removed, and the biofilms were washed three times with PBS and air-dried at room temperature for 20 min. For EPS quantification, 40 μL of distilled water, 40 μL of 6% phenol solution, and 200 μL of 97% sulfuric acid were sequentially added to each well, and OD₄₉₀ was measured after allowing the reaction for 30 min at room temperature.

Biofilms were cultured by inoculating 250 μL of bacterial suspension with an equal volume of 1% BHIS broth in 24-well plates, with or without PMMA-MPs. Each concentration was prepared in triplicate. Following 24 h incubation at 37 °C, the cultures were centrifuged (6,000 rpm, 10 min, 4 °C), and supernatant was collected. Lactic acid production was quantified using a D-Lactate Assay Kit with WST-8 (S0204S, Beyotime, Shanghai, China) following the manufacturer’s instructions. Simultaneously, the pH of the supernatant was measured using a calibrated pH meter (PHS-3E, Leici, Shanghai, China).

### 2.7 Antibiotic tolerance assessment

The effect of PMMA-MPs on bacterial antibiotic tolerance was evaluated using chlorhexidine digluconate (CHG), minocycline hydrochloride (MH), and cefazolin (CZO). Bacterial suspensions were inoculated into 96-well plates containing 1% BHIS broth, supplemented with varying concentrations of PMMA-MPs (15, 30, and 60 μg/mL) and antibiotics. The control group consisted of 1% BHIS broth without antibiotics. Each condition was tested in triplicate. The antibiotic concentrations were as follows: CHG, 0.25–32 μg/mL; MH, 0.25–32 μg/mL; and CZO, 0.03125–8 μg/mL. OD₆₀₀ was measured after 24 h of incubation. Cell viability was expressed as the percentage of OD values relative to the control group. Dose–response curves were fitted, and the half-maximal inhibitory concentration (IC₅₀) was calculated using GraphPad Prism 9.

### 2.8 Transcriptome analysis

A 500 μL bacterial suspension was mixed with an equal volume of 1% BHIS broth in 6-well plates, with or without 15 μg/mL PMMA-MPs. Each condition was tested in triplicate. Following 24 h of incubation at 37 °C, the medium was replaced with fresh 1% BHIS broth, and the biofilms were allowed to mature for an additional 24 h. The mature biofilms were then centrifuged (6,000 rpm, 10 min, 4 °C), and the resulting pellet was collected for subsequent analysis. Total RNA was isolated using the Trizol Reagent (Invitrogen Life Technologies). Quality and integrity were determined using a NanoDrop spectrophotometer (Thermo Scientific) and a Bioanalyzer 2,100 system (Agilent). Zymo-Seq RiboFree Total RNA Library Kit was used to remove rRNA from total RNA. Random oligonucleotides and SuperScript III were used to synthesize the first strand cDNA. Remaining overhangs were converted into blunt ends via exonuclease/polymerase activities and the enzymes were removed. After adenylation of the 3′ ends of the DNA fragments, Illumina PE adapter oligonucleotides were ligated to prepare for hybridization. To select cDNA fragments of the preferred 400–500 bp in length, the library fragments were purified using the AMPure XP system (Beckman Coulter, Beverly, CA, United States). DNA fragments with ligated adaptor molecules on both ends were selectively enriched using Illumina PCR Primer Cocktail in a 15 cycle PCR reaction. Products were purified (AMPure XP system) and quantified using the Agilent high sensitivity DNA assay on a Bioanalyzer 2,100 system (Agilent). The sequencing library was then sequenced on NovaSeq 6,000 platform (Illumina). The data were analyzed by using the online platform Personalbio GenesCloud (see Footnote 1). The expression of differentially expressed genes (DEGs) was evaluated by qRT-PCR. The experimental approach is the same as in transcriptome analysis. Three independent experiments were performed. The primer sequences used in the experiment are listed in Supplementary Table S1.

### 2.9 Statistical analysis

Data were expressed as mean ± standard deviation, derived from three independent biological experiments, each performed with three technical replicates. GraphPad Prism 9 was used for data analysis and graph plotting. The statistical methods employed for each experiment are described in the corresponding figure legends. Statistical significance was set at *p* < 0.05.

## 3 Results

### 3.1 Characterization of PMMA-MPs

**Figure 1:**
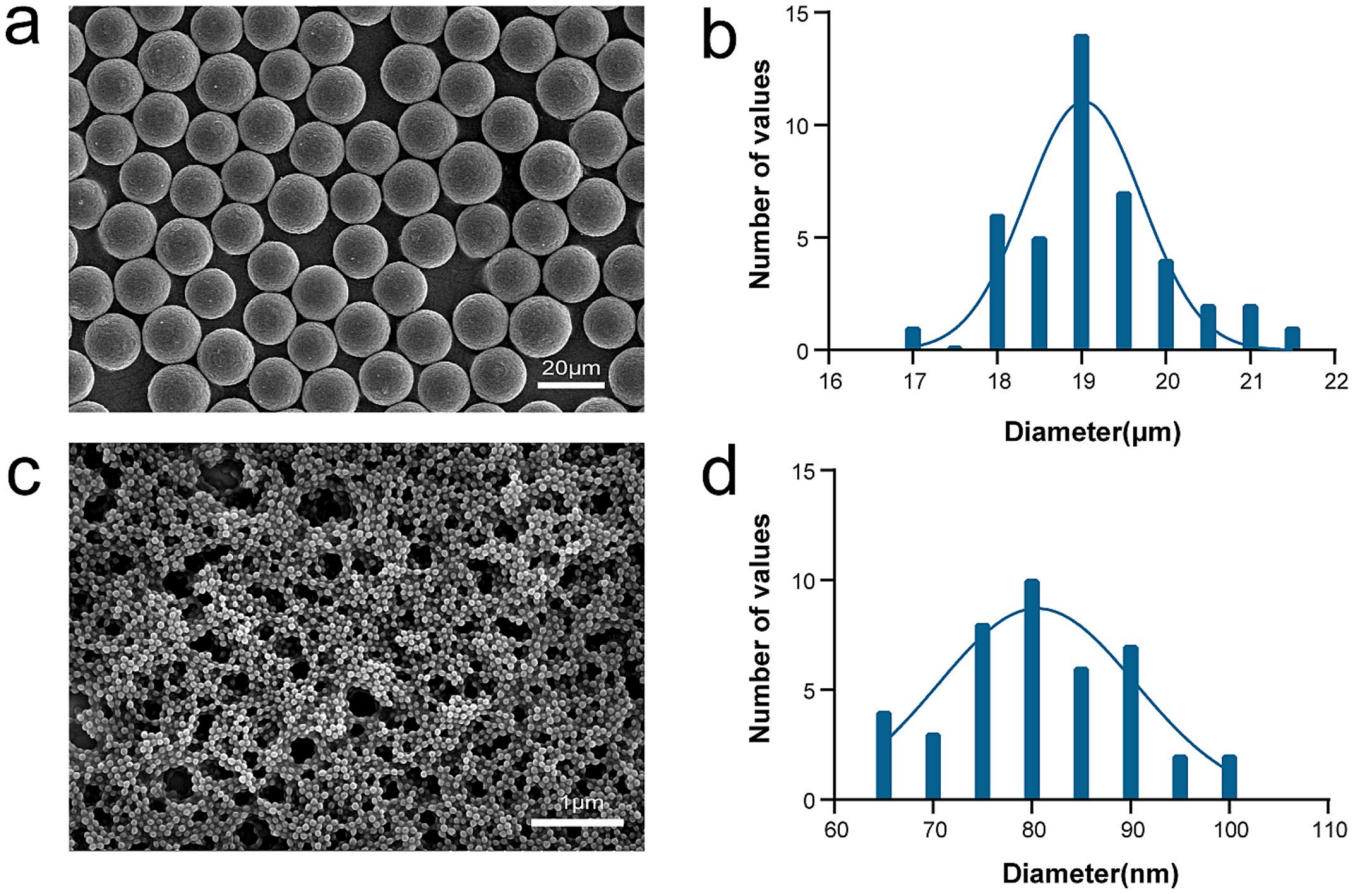
Characterization of PMMA-MPs. **(a)** SEM image of 20 μm PMMA-MPs. **(b)** Particle dimensions of 20 μm PMMA-MPs. **(c)** SEM image of 80 nm PMMA-NPs. **(d)** Particle dimensions of 80 nm PMMA-NPs.

The SEM characterization of PMMA-MPs with sizes of 80 nm and 20 μm revealed spherical morphology, with particle dimensions consistent with manufacturer specifications (Figure 1). Zeta potential measurements in BHI medium revealed surface charge values of ˗11.26 and ˗27.47 mV for 80 nm and 20 μm particles, respectively.

### 3.2 PMMA-MPs altered oral microbiota composition

**Figure 2:**
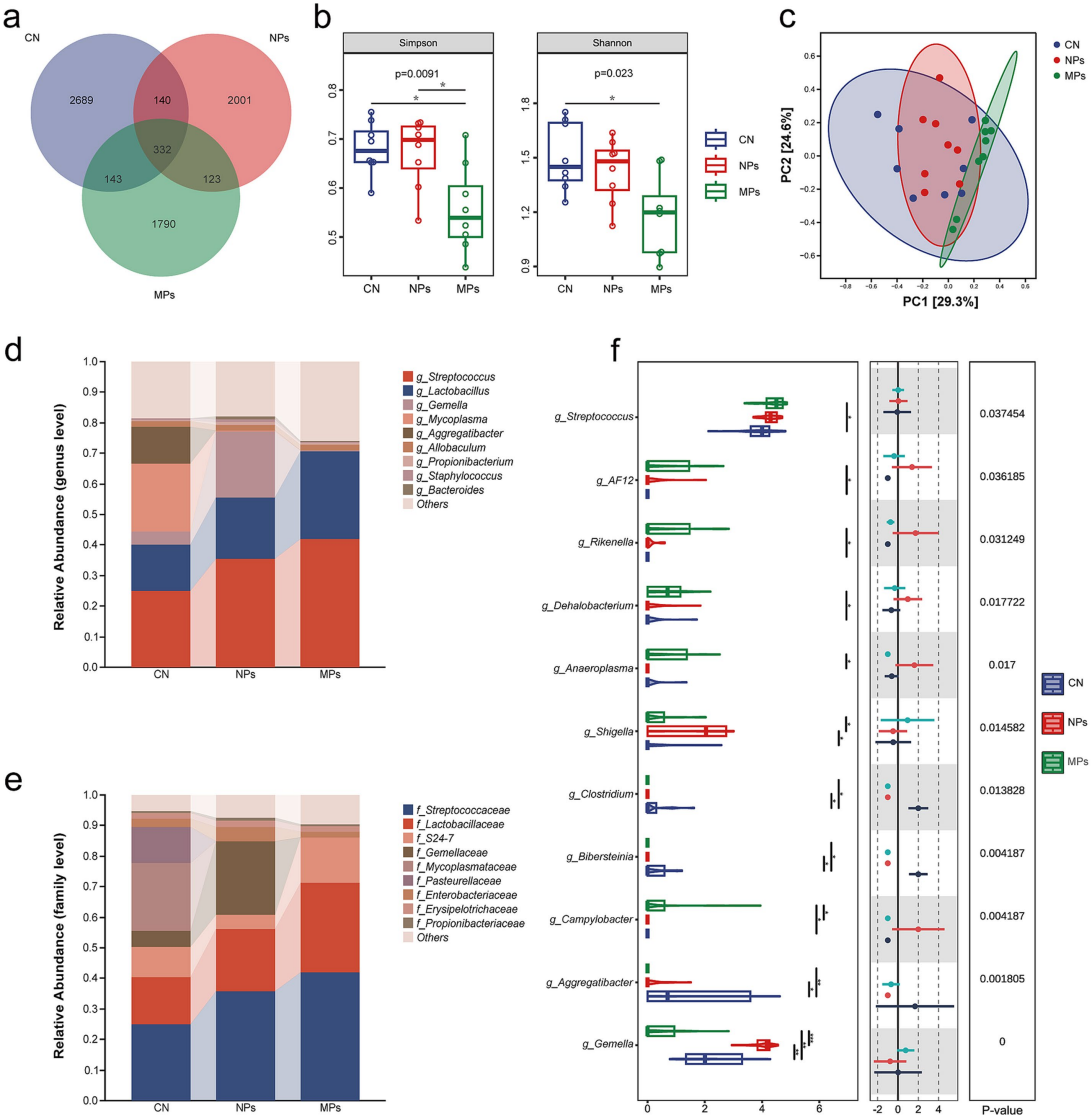
Effect of PMMA-MPs on oral microbiota dysbiosis in mice. **(a)** Venn diagram showing the average numbers of OTUs in different groups. **(b)** α-Diversity (Simpson and Shannon) of each group; each box plot represents the median, interquartile range, minimum, and maximum values. **(c)** Principal coordinate analysis of each group. **(d)** Composition of the oral microbiota at the genus level in each group. **(e)** Composition of the oral microbiota at the family level in each group. **(f)** Taxonomic differences analysis of each group. The Kruskal–Wallis *H* test was used to assess overall differences among multiple groups. Following a significant Kruskal–Wallis result, Dunn‘s test with Bonferroni adjustment was performed for *post hoc* pairwise comparisons. *p < 0.05.

**Figure 3:**
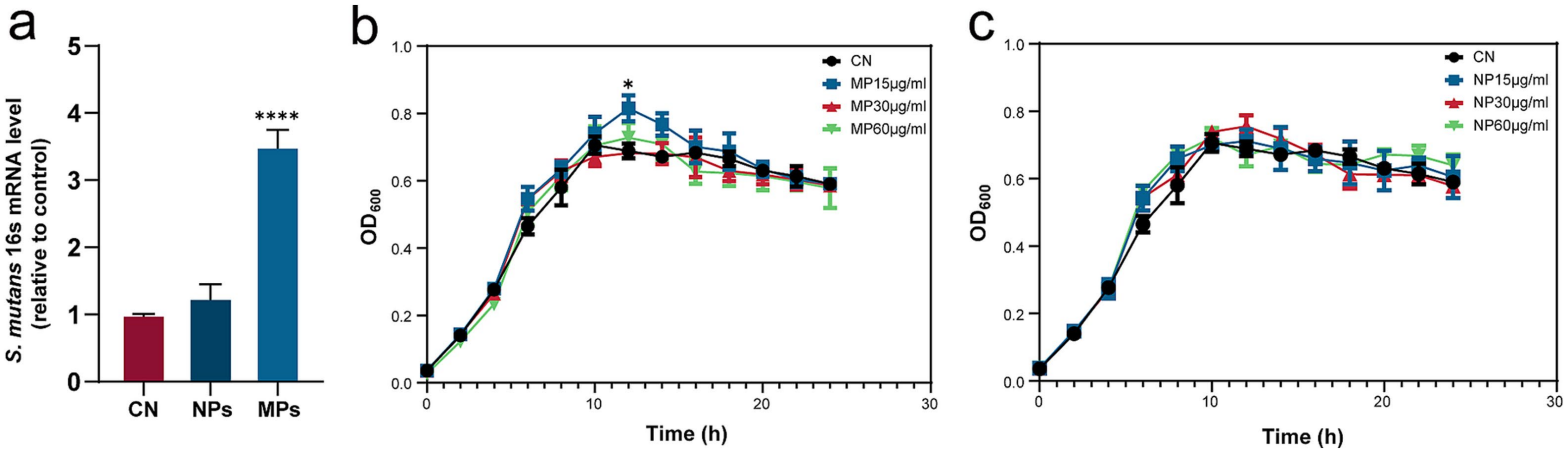
Results of qRT-PCR to examine the specific genes of *S. mutans* **(a)** and growth curve of *S. mutans* with PMMA-MPs **(b)** or PMMA-NPs **(c)**. Statistical significance was determined by one-way ANOVA with Dunnett’s *post hoc* test **(a)** or two-way ANOVA with Bonferroni correction **(b,c)**. *p < 0.05 and ****p < 0.0001.

The oral microbiota composition across experimental groups was assessed through 16S rRNA sequencing of mouse oral swab samples. Venn diagram analysis illustrated the average number of shared operational taxonomic units (OTUs) among the CN, MPs, and NPs groups (Figure 2a). The MPs group showed significantly altered *α*-diversity indices (Simpson and Shannon) compared to the other two groups (Figure 2b). Principal coordinate analysis demonstrated that PMMA-MP exposure altered the overall microbial composition relative to the CN group, with notable shifts in the oral microbiota community. In contrast, the NPs group exhibited a microbial profile similar to the CN group (Figure 2c). At the taxonomic level, *Streptococcus* and *Streptococcaceae* were the most abundant genus and family, respectively, across all groups (Figures 2d,e). Both the NPs and MPs groups demonstrated an increasing trend in the relative abundance of *Streptococcus* and Streptococcaceae, with the MPs group displaying a statistically significant elevation in *Streptococcus* abundance compared to the CN group (*p* = 0.037) (Figure 2f). Subsequently, we examined the expression of *S. mutans*-specific genes in each sample group using qRT-PCR. The results showed that the expression of specific genes was significantly upregulated in the MPs group (*p* < 0.0001) (Figure 3a).

### 3.3 Effects of PMMA-MPs on cell viability

To investigate the influence of PMMA-MPs on bacterial growth, *S. mutans* was exposed to a concentration gradient of PMMA-MPs for 24 h. *S. mutans* demonstrated rapid growth during the first 10 h, reaching the peak OD₆₀₀ (0.7058), followed by gradual growth stabilization (Figures 3b,c). Notably, the MPs group with 15 μg/mL concentration demonstrated accelerated growth kinetics (*p* = 0.0426), achieving maximum OD₆₀₀ (0.8152) at 12 h. No significant differences in growth patterns were observed between other treatment groups and the CN control group.

### 3.4 PMMA-MPs induced enhanced biofilm formation

**Figure 4:**
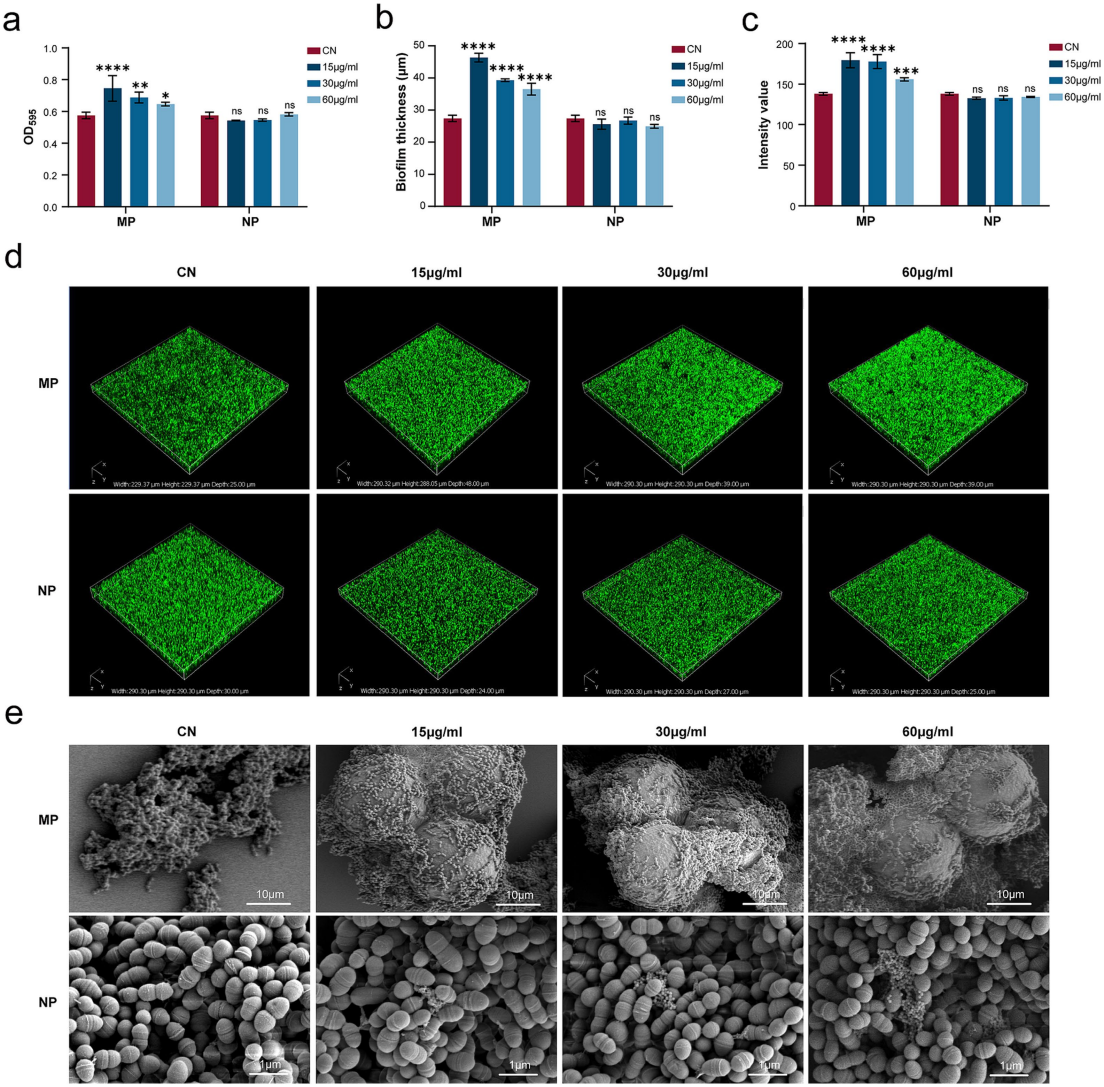
*Streptococcus mutans* biofilm formation following PMMA-MP exposure at different concentrations. **(a)** Crystal violet staining of biofilm. **(b)** Biofilm thickness. **(c)** Intensity value. **(d)** CLSM observation of biofilm. **(e)** SEM images of *S. mutans*. Statistical significance was determined by two-way ANOVA with Bonferroni correction. *p < 0.05, **p < 0.01, ***p < 0.001, and ****p < 0.0001.

*Streptococcus mutans* biofilm-forming capability following PMMA-MPs exposure was quantitatively assessed using crystal violet staining. PMMA-MPs exposure significantly enhanced biofilm formation after 24 h incubation (Figure 4), with the most pronounced effect observed at 15 μg/mL (Figure 4a). In contrast, no significant alterations in biofilm formation were detected in PMMA-NPs groups. Bacterial biofilm architecture, including three-dimensional structure and thickness, was visualized by CLSM. Three-dimensional CLSM reconstructions revealed sparse and heterogeneous biofilm structures in the CN, whereas PMMA-MP exposure resulted in markedly thicker and more compact biofilms (Figure 4d). Z-section analysis demonstrated a substantial increase in average biofilm thickness from 27.41 ± 2.36 μm (CN) to 46.35 ± 3.18 μm (15 μg/mL PMMA-MPs) (Figure 4b). Fluorescence intensity analysis further confirmed enhanced biofilm matrix production at 15 μg/mL PMMA-MPs, showing significantly higher values compared to controls (*p* < 0.0001) (Figure 4c), consistent with the crystal violet quantification results. SEM analysis revealed that PMMA-MP particles served as additional colonization sites for bacterial adhesion, becoming extensively embedded within extracellular polymeric substances and bacterial clusters (Figure 4e). Biofilms in the MPs group exhibited more uniform and densely packed architectures than in the CN group. Notably, PMMA-NPs exposure did not induce discernible structural damage to bacterial cell walls or significantly modify biofilm integrity. Based on these findings, PMMA-MPs was selected for subsequent investigation of their bacteriological effects.

### 3.5 Effects of PMMA-MPs on EPS and lactic acid production

**Figure 5:**
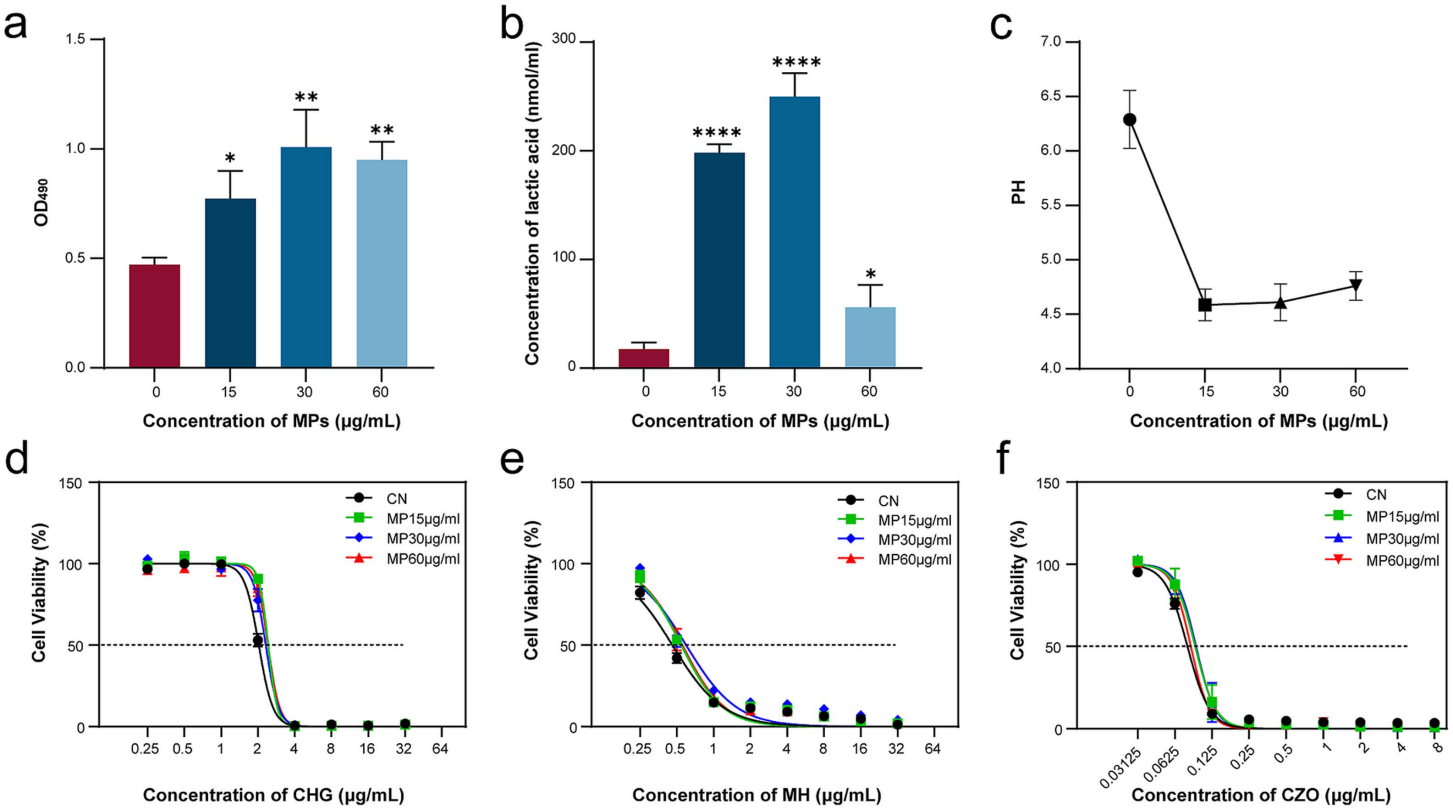
Effects of PMMA-MPs on EPS, lactic acid production, and antibiotic tolerance. **(a)** EPS; **(b)** Lactic acid; **(c)** pH value. Dose–response relationship of bacterial growth following PMMA-MP and antibiotic exposure: **(d)** CHG; **(e)** MH; **(f)** CZO. Statistical significance was determined by one-way ANOVA with Dunnett’s *post hoc* test. *p < 0.05, **p < 0.01, and ****p < 0.0001.

EPS, the primary structural component of bacterial biofilms, was quantitatively evaluated to elucidate the mechanism underlying PMMA-MPs-enhanced biofilm formation. PMMA-MP exposure significantly increased EPS production by *S. mutans* at all tested concentrations compared to the CN (Figure 5a). The acid-producing capacity of PMMA-MP-exposed bacteria was investigated through lactate quantification and pH measurements. PMMA-MPs treatment enhanced lactate production, and the most pronounced effects were observed at 15 and 30 μg/mL (Figure 5b). Correspondingly, the culture pH decreased from 6.9 (CN) to 4.59 (15 μg/mL) (Figure 5c).

### 3.6 PMMA-MPs induced enhanced antibiotic tolerance

Treatment with PMMA-MPs at varying concentrations resulted in elevated IC₅₀ values (concentration required for 50% growth inhibition) for all tested antimicrobials (CHG, MH, and CZO) compared to the control group (Figures 5d–f). Notably, PMMA-MP exposure at 15 μg/mL increased the IC₅₀ for CHG, MH, and CZO from 2.033 to 2.429 μg/mL, 0.4595 to 0.5423 μg/mL, and 0.07906 to 0.09137 μg/mL (Table 1), respectively, demonstrating consistent enhancement of microbial tolerance across different drug classes.

**Table 1:** Effects of PMMA-MPs on antibiotic tolerance of *S. mutans*.

Antibiotic types	Half inhibitory concentration (IC50, 95%CI, μg/mL)			
	CN	15 μg/mL MPs	30 μg/mL MPs	60 μg/mL MPs
CHG	2.033 2.000–2.067 *R*^2^ = 0.9981	2.429 1.962–3.007 *R*^2^ = 0.9982	2.319 2.116–2.542 *R*^2^ = 0.9962	2.416 2.159–2.703 *R*^2^ = 0.9962
MH	0.4595 0.4173–0.5063 *R*^2^ = 0.9561	0.5423 0.4948–0.5963 *R*^2^ = 0.9625	0.5948 0.5116–0.7007 *R*^2^ = 0.9192	0.5544 0.5025–0.6138 *R*^2^ = 0.9585
CZO	0.07906 0.07521–0.08312 *R*^2^ = 0.9877	0.09137 0.08606–0.09703 *R*^2^ = 0.9872	0.09268 0.08702–0.09887 *R*^2^ = 0.9866	0.08418 0.08088–0.08753 *R*^2^ = 0.9944

CN, control group; MPs, microplastics; CHG, chlorhexidine digluconate; MH, minocycline hydrochloride; CZO, cefazolin.

### 3.7 Transcriptome analysis of DEGs

**Figure 6:**
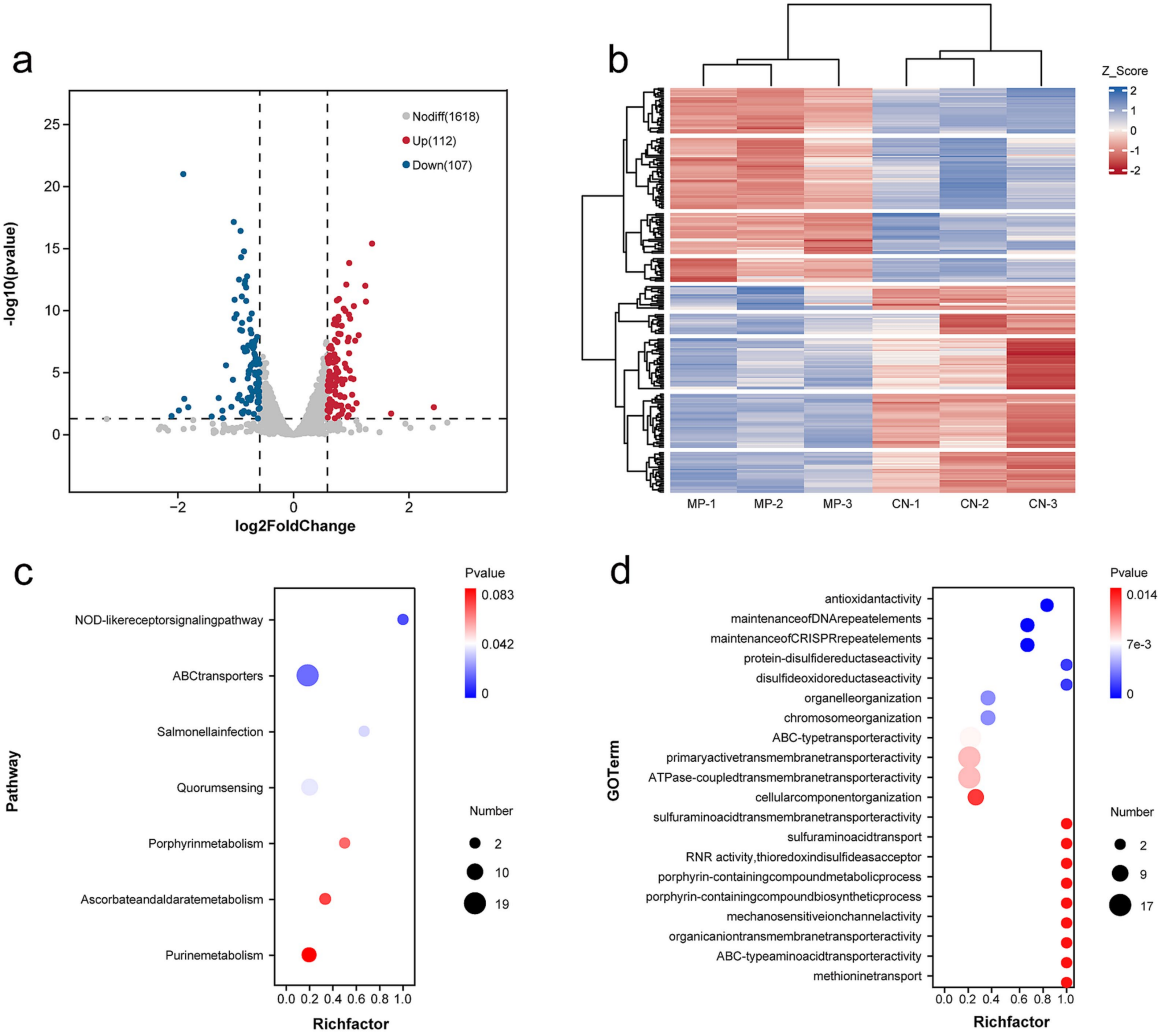
Transcriptome analysis of DEGs following PMMA-MPs exposure. **(a)** Volcano map of all DEGs. Red plots represent upregulated genes. Blue plots represent downregulated genes. Grey plots represent normally expressed mRNAs. **(b)** Heatmap of all DEGs. The horizontal axis represents the sample, and the vertical axis represents different genes. The red color indicates increased gene expression, and the blue indicates decreased gene expression. **(c)** KEGG enrichment analysis of DEGs. **(d)** GO enrichment analysis of DEGs.

**Figure 7:**
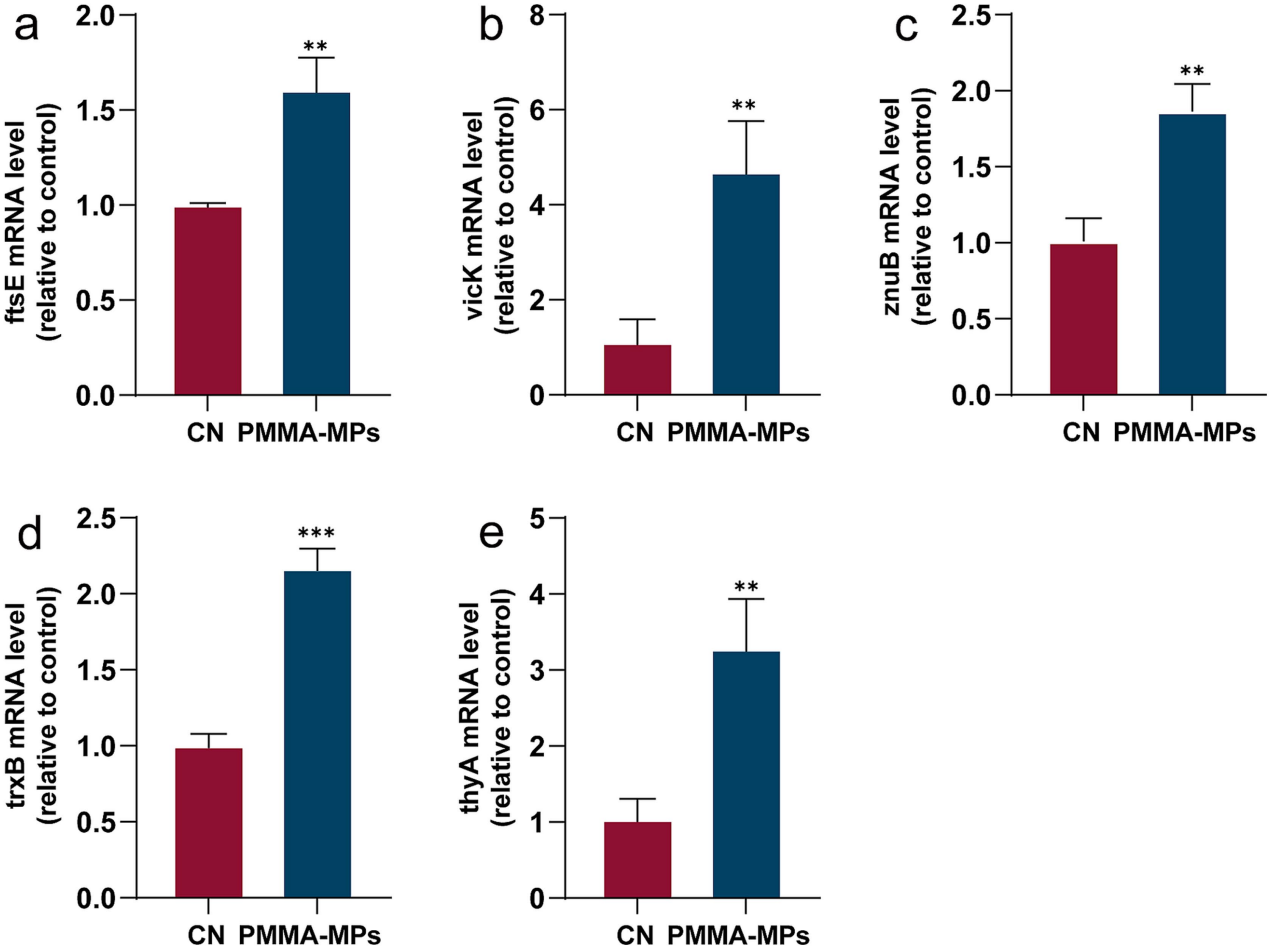
Results of qRT-PCR to validate transcriptomic DEGs, including ftsE **(a)**, vicK **(b)**, znuB **(c)**, trxB **(d)**, and thyA **(e)**. Results are expressed using the 2^−ΔΔCt^ method as mean ± SD. Statistical comparisons between the two groups were performed using an unpaired, two-tailed Student’s *t*-test. ^**^p < 0.01 and ^***^p < 0.001.

Given that 15 μg/mL PMMA-MPs induced the most pronounced enhancement of bacterial biofilm, transcriptome sequencing was performed to analyze genome-wide changes at this concentration. DEGs between the control and MPs groups were identified using an adjusted *p* < 0.05. The volcano plot and heatmap (Figures 6a,b) revealed 112 up-regulated and 107 down-regulated genes. We summarized the key DEGs based on the sequencing results. As shown in Supplementary Table S2, the expression levels of *ftsE*, *vicK*, *znuB*, *trxB*, and *thyA* were upregulated following PMMA-MPs exposure. qRT-PCR was then performed to validate the expression trends, and the results are presented in Figure 7. Functional annotation of DEGs was performed through Kyoto Encyclopedia of Genes and Genomes (KEGG) and Gene Ontology (GO) enrichment analyses. KEGG pathway analysis identified seven significantly enriched pathways, among which ABC transporters, quorum sensing, and purine metabolism were critically associated with bacterial biofilm formation (Figure 6c). GO analysis revealed the enrichment of most DEGs in biofilm development, primarily through mechanisms involving bacterial adhesion, EPS synthesis, oxidative stress resistance, and quorum sensing (Figure 6d).

## 4 Discussion

### 4.1 Alterations in oral microbiome profiles following PMMA-MPs exposure

The oral cavity harbors the most dense and diverse microbial communities in the human body, continuously exposed to dietary and environmental factors. Perturbations in the oral microbiota have been strongly associated with various diseases, including periodontitis, dental caries, and oral cancer (Kilian, 2018). As the gateway to both the digestive and respiratory tracts, the oral microbiome significantly influences downstream microbial colonization patterns (Sedghi et al., 2021). Additionally, the highly vascularized oral mucosa facilitates systemic translocation of oral microbes, implicating oral dysbiosis in multiple systemic diseases (Hajishengallis and Chavakis, 2021). While previous studies have focused on the ecotoxicology of MPs in gut microbiota (Zheng et al., 2025; Chen et al., 2022; Zhang et al., 2024; Jin et al., 2019), their impact on the oral niche—a unique ecosystem characterized by nonsterile conditions and constant exposure to exogenous materials—remains largely unexplored. This study provides the first evidence that PMMA-MPs induce alterations in the oral microbial community by murine exposure model.

PMMA-MPs exposure induced significant shifts in the murine oral microbiota composition at both the family and genus levels. The NPs group exhibited a marked increase in the abundance of *Shigella*, a primary pathogen responsible for shigellosis and diarrhea (Scott et al., 2025). The MPs group exhibited distinct *α*-diversity and microbial profiles compared to other groups. Based on published literature, PMMA-MNPs may exert physical and chemical pressures on the oral microbial community, leading to alterations in its composition. These pressures may arise from direct particle-microbe interactions, including membrane disruption, surface adhesion, and nanoparticle internalization, as well as from the leaching of residual monomers or additives and changes in the local microenvironment (Liu et al., 2025; Boháčková and Cajthaml, 2024).

Eleven bacterial taxa showed significant alterations upon PMMA-MPs exposure. Among these, *Campylobacter*, *Anaeroplasma*, and *Streptococcus* were substantially enriched in the MPs group relative to the CN controls. *Campylobacter* has been implicated in inflammatory bowel disease (IBD), with intestinal invasive strains detected in 50% of patients with IBD but not in healthy individuals (Castaño-Rodríguez et al., 2017). *Streptococcus* are dominant species in saliva and oral soft tissues, with most oral habitats being primarily colonized by *Streptococcus* (Abranches et al., 2018). Oral *Streptococcus* have been classified into the following eight groups: mitis, sanguinis, anginosus, salivarius, downei, mutans, pyogenic, and bovis (Richards et al., 2014). Many streptococcal species produce abundant adhesive molecules that enable effective colonization at diverse oral sites. Among them, *S. mutans* is a well-established cariogenic pathogen and possesses the capacity for systemic dissemination, which may lead to intestinal colonization (exacerbating colitis), bacteremia, or infective endocarditis (Peng et al., 2022). Therefore, PMMA-MPs may disrupt oral microbial homeostasis and elevate susceptibility to various bacterial infections. Of particular concern was the consistent enrichment of *Streptococcus* across both the MPs and NPs groups. The hydrophobic surface of PMMA-MPs provides additional binding sites for *Streptococcus*, further amplifying their colonization advantage. However, whether the presence of PMMA-MPs exerts selective toxicity against other microbial taxa, thereby reducing microbial competition, requires further validation through integrated transcriptomic, proteomic, and metabolomic analyses in future studies.

### 4.2 Mechanisms underlying PMMA-MPs-induced *S. mutans* biofilm formation

The PMMA is one of the key constituent materials used in dental restorative resins and dentures. *S. mutans* exhibits a pronounced ability to form biofilms on solid surfaces, such as tooth enamel, dentures, and dental restorative resins, showing more extensive accumulation on resin-based composites than on enamel or other prosthetic materials (Svanberg et al., 1990). This biofilm development consequently elevates the risk of secondary caries formation. These structured biofilms form complex three-dimensional architectures that enhance bacterial tolerance to antibiotics and environmental stresses while increasing pathogenicity (Lemos et al., 2019; Guo et al., 2023).

In the present study, quantitative biofilm assays revealed a significant increase in *S. mutans* biofilm biomass following PMMA-MPs exposure. CLSM further demonstrated that PMMA-MPs-treated bacteria developed thicker and more compact biofilms. These observations are consistent with those of Li et al. (2023), who reported preferential bacterial colonization on MPs surfaces enhanced by EPS-mediated adhesion. This phenomenon may be driven by the unique physicochemical properties of MPs. Several studies have reported microbial predilection for hydrophobic MPs surfaces with strong interfacial interactions (Krasowska and Sigler, 2014). Furthermore, the characteristic surface roughness, high porosity, and large specific surface area of MPs offer optimal conditions for bacterial colonization and community development (Dong et al., 2020). Therefore, we reasonably hypothesize that one potential mechanism underlying the increased biofilm biomass of *S. mutans* following PMMA-MPs exposure is that PMMA-MPs may provide an additional hydrophobic surface for bacterial adhesion, thereby triggering the expression of biofilm-associated genes.

We performed transcriptomic analysis of *S. mutans* following PMMA-MPs exposure to elucidate the mechanisms underlying PMMA-MPs-enhanced biofilm formation. The DEGs were associated with several key pathways, including the NOD-like receptor signaling pathway, ABC transporters, *Salmonella* infection, quorum sensing, porphyrin metabolism, ascorbate and aldarate metabolism, and purine metabolism. Among these, the ABC transporter and quorum sensing pathways are particularly critical for biofilm development (Li et al., 2018; Imchen et al., 2022). The ABC transporter system facilitates the uptake of carbon sources essential for bacterial ATP production, cellular component synthesis, and biofilm matrix formation, such as sucrose and fructose (Ma et al., 2025). Quorum sensing, a cell-to-cell communication mechanism, regulates biofilm formation by modulating extracellular matrix production (Imchen et al., 2022). Transcriptomic data revealed upregulation of *ftsE*, a gene within the ABC transporter system that controls EPS secretion. We also observed a significant upregulation of *vicK*, a histidine kinase (part of the VicRK two-component system) that directly regulates biofilm formation, EPS synthesis, and acid tolerance in *S. mutans* (Senadheera et al., 2007). These findings collectively suggest that PMMA-MPs promote biofilm formation by activating multiple interconnected pathways involved in carbohydrate metabolism, cell signaling, and stress adaptation.

### 4.3 Mechanisms of PMMA-MPs in enhancing bacterial pathogenicity

*Streptococcus mutans* possesses a complete glycolytic pathway capable of metabolizing diverse carbohydrates to produce lactic acid and other fermentation byproducts, thereby lowering the environmental pH (Ajdić et al., 2002). Unlike other acidogenic bacteria, *S. mutans* uniquely utilizes dietary sugars to synthesize an insoluble EPS-enriched biofilm matrix—a key virulence trait (Klein et al., 2015). PMMA-MPs significantly enhanced both EPS production and lactic acid secretion, concomitant with a marked pH reduction in bacterial cultures, findings consistent with the quantitative biofilm measurements. Notably, even low PMMA-MPs exposure substantially increased tolerance to all three tested antibiotics. Transcriptomic analysis revealed upregulation of antibiotic tolerance-associated genes (*znuB*, *trxB*, *thyA*) and related pathways, consistent with the findings of Brazel et al. (2022) and Lu and Holmgren (2014). Based on other published studies on bacterial tolerance (Ławniczak et al., 2011; Hirt and Body-Malapel, 2020; Stevenson et al., 2024), we reasonably hypothesize that the following three synergistic mechanisms may underlie this phenotypic adaptation: (i) Biofilm-mediated protection: the structured biofilm matrix reduces antibiotic penetration and enhances biochemical tolerance through quorum sensing-mediated bacterial communication. (ii) Horizontal gene transfer facilitation: reduced intercellular spacing within biofilms accelerates plasmid DNA exchange compared to planktonic conditions. (iii) Antibiotic adsorption: The high binding affinity of PMMA-MPs may deplete free antibiotic concentration in the aqueous phase while concentrating the drugs on particle surfaces, limiting bactericidal exposure. However, the emergent tolerance phenotype is likely driven by a combination of these mechanisms, and further investigation is warranted to delineate their relative contributions.

Integrating these findings, we propose a multi-stage mechanism by which PMMA-MPs enhance the pathogenicity of *S. mutans*. The hydrophobic surface and high porosity of PMMA-MPs promote initial bacterial adhesion and biofilm nucleation. This physical interaction may act as an environmental signal, activating signal transduction systems (e.g., the VicRK two-component system and quorum sensing pathways), which subsequently orchestrate a metabolic shift involving upregulated EPS production, heightened acidogenicity, and elevated antibiotic tolerance.

### 4.4 Limitations

This study had some limitations. First, the PMMA-MPs used were of industrial-grade standardized products, which may not fully replicate the diverse shapes and aging degrees of MPs released from PMMA due to physiological oral activities in humans. Additionally, the wear of dental restorative materials or resin-based dentures can release other particles or compounds, such as inorganic fillers, methyl methacrylate, and methacrylic acid. However, this study did not investigate the combined effects of PMMA-MPs with these additional components. Furthermore, the oral environment is highly dynamic, influenced by mastication, swallowing, and salivary flow, all of which can lead to continuous fluctuations in PMMA-MPs concentrations. The static concentrations employed in this study may not accurately reflect real-world exposure levels. Therefore, future research should incorporate clinical samples and consider broader concentration ranges in both *in vivo* and *in vitro* experiments, ensuring greater ecological relevance.

## 5 Conclusion

This study systematically investigated the effects of PMMA-MPs on *S. mutans* biofilm formation, virulence, and antibiotic tolerance. PMMA-MPs significantly altered the oral microbiota composition in mice, with a marked increase in *Streptococcus* abundance. *In vitro* experiments revealed that PMMA-MPs exposure enhanced *S. mutans* growth, biofilm biomass, and structural density. Notably, PMMA-MPs also exacerbated the pathogenicity of *S. mutans* by promoting EPS production, lactic acid secretion, and acidification of the microenvironment. These in vitro findings suggest that PMMA-MPs may contribute to the caries process indirectly by influencing bacterial pathogenicity. However, as this study did not directly assess enamel demineralization or carious lesion formation, further validation using tooth hard tissue models or animal studies is required to confirm their actual cariogenic effect. This study has broad implications across multiple fields. From an environmental toxicology perspective, it identifies the oral cavity as a critical yet long-overlooked entry portal for microplastics, where they directly interact with the oral microbiota prior to systemic translocation. This interaction has implications for oral health, as PMMA-MPs enhance the enrichment and pathogenicity of *S. mutans*. Furthermore, the increased antibiotic tolerance observed in bacterial biofilms exposed to microplastics raises public health concerns, as microplastics may facilitate horizontal gene transfer, potentially turning the oral microbiome into a reservoir for antimicrobial resistance genes. Collectively, these findings underscore the urgent need to re-evaluate the long-term safety of polymer-based dental materials.

## Data Availability

The data presented in the study are deposited in the NCBI Sequence Read Archive (SRA) database under accession numbers PRJNA1441217 and PRJNA1442144.

## Footnotes

1
https://www.genescloud.cn/
