# Supplementary material for: Polymethyl methacrylate microplastics affect oral microbiota diversity and Streptococcus mutans biofilm formation
Source: Front Microbiol. 2026 Apr 14;17:1811667. doi: 10.3389/fmicb.2026.1811667 (PMC13121379; doi:10.3389/fmicb.2026.1811667)
Supplement: Supplementary file 2 [file Supplementary_file_1.docx]

| Primers | | Sequences (5' to 3') |
| --- | --- | --- |
| *16s rRNA* of oral microbiota | Forward | GTGCCAGCMGCCGCGGTAA |
|  | Reverse | CCGTCAATTCMTTTRAGTTT |
| *16s rRNA* of  *S. mutans* | Forward | AGCGTTGTCCGGATTTATTG |
|  | Reverse | CTACGCATTTCACCGCTACA |
| *ftsE* | Forward | GAAATTCCCATCCTGCGTCG |
|  | Reverse | ATGGCAACAACACGATGACG |
| *vicK* | Forward | TGATGCGACCGAACAGGAAA |
|  | Reverse | AGCACGGCTTCTTGCCTTAT |
| *znuB* | Forward | AGCAGGTGTTGCTTTTGGTG |
|  | Reverse | ACGAACTGGCAAACCATCCA |
| *trxB* | Forward | TTGCTGTCGCTGGTGAAGAA |
|  | Reverse | ATAGCAAAAACGCCTGCTGC |
| *thyA* | Forward | TGATGGTGTCTGGAGCGAAC |
|  | Reverse | TATCGTTGGAGCGCTGTGTT |

Table S1. Primer sequences used in this study

Table S2. Differentially expressed genes

| id | Name | Annotation | pval | padj | Regulation |
| --- | --- | --- | --- | --- | --- |
| gene-I6L88_RS02930 | ftsE | cell division protein FtsE | 1.532e-06 | 3.065e-05 | Up |
| gene-I6L88_RS02135 | vicK | cell wall metabolism sensor histidine kinase VicK | 1.129e-06 | 2.393e-05 | Up |
| gene-I6L88_RS09955 | znuB | zinc ABC transporter permease subunit ZnuB | 0.0001077 | 0.001015 | Up |
| gene-I6L88_RS06760 | trxB | thioredoxin reductase | 4.9e-10 | 2.847e-08 | Up |
| gene-I6L88_RS04600 | thyA | thymidylate synthase | 1.656e-09 | 8.282e-08 | Up |

pval: p-value, padj: p-adjust value.
